# A Case of AML Characterized by a Novel t(4;15)(q31;q22) Translocation That Confers a Growth-Stimulatory Response to Retinoid-Based Therapy

**DOI:** 10.3390/ijms18071492

**Published:** 2017-07-11

**Authors:** Justin M. Watts, Aymee Perez, Lutecia Pereira, Yao-Shan Fan, Geoffrey Brown, Francisco Vega, Kevin Petrie, Ronan T. Swords, Arthur Zelent

**Affiliations:** 1 Division of Hematology/Oncology, Department of Medicine, Miller School of Medicine, University of Miami, Miami, FL 33136, USA; jxw401@miami.edu (J.M.W.); aperez10@med.miami.edu (A.P.); lpereira@med.miami.edu (L.P.); rswords@med.miami.edu (R.T.S.); 2 Department of Pathology, Miller School of Medicine, University of Miami, Miami, FL 33136, USA; yfan@med.miami.edu (Y.-S.F.); fvega@med.miami.edu (F.V.); 3 Institute of Immunology and Immunotherapy, University of Birmingham, Birmingham B15 2TT, UK; g.brown@bham.ac.uk; 4 Institute of Clinical Sciences, University of Birmingham, Birmingham B15 2TT, UK; 5 Faculty of Natural Sciences, University of Stirling, Stirling FK9 4LA, UK

**Keywords:** acute myeloid leukemia, t(4, 15)(q31, q22), TMEM154-RASGRF1, ATRA, retinoid, NCT02273102

## Abstract

Here we report the case of a 30-year-old woman with relapsed acute myeloid leukemia (AML) who was treated with all-*trans* retinoic acid (ATRA) as part of investigational therapy (NCT02273102). The patient died from rapid disease progression following eight days of continuous treatment with ATRA. Karyotype analysis and RNA-Seq revealed the presence of a novel t(4;15)(q31;q22) reciprocal translocation involving the *TMEM154* and *RASGRF1* genes. Analysis of primary cells from the patient revealed the expression of *TMEM154-RASGRF1* mRNA and the resulting fusion protein, but no expression of the reciprocal *RASGRF1-TMEM154* fusion. Consistent with the response of the patient to ATRA therapy, we observed a rapid proliferation of t(4;15) primary cells following ATRA treatment ex vivo. Preliminary characterization of the retinoid response of t(4;15) AML revealed that in stark contrast to non-t(4;15) AML, these cells proliferate in response to specific agonists of RARα and RARγ. Furthermore, we observed an increase in the levels of nuclear RARγ upon ATRA treatment. In summary, the identification of the novel t(4;15)(q31;q22) reciprocal translocation opens new avenues in the study of retinoid resistance and provides potential for a new biomarker for therapy of AML.

## 1. Introduction

Acute myeloid leukemia (AML) is the most commonly occurring leukemia in adults, accounting for an estimated 33% of all cases and more that 80% of acute cases in 2016 [1]. AML is a disease whose incidence increases with age and—in line with an aging population—this has increased from 3.4 per 100,000 in 2004 to 5.1 per 100,000 in 2013 [2]. Advances in the treatment of AML have dramatically improved treatment outcomes for younger patients, with a relative 5-year survival rate (2005–2011) of approximately 67% (0–14 years) [1]. In the elderly, however (who account for the majority of new cases), the prognosis remains poor. Indeed, AML accounts for an estimated 43% of all leukemia deaths, with an overall average 5-year survival rate of only 27% [2].

The use of combination therapy based on the use all-*trans* retinoic acid (ATRA) and arsenic trioxide (As_2_O_3_) has revolutionized the clinical outcome of acute promyelocytic leukemia (APL), with complete remission rates of 90% and cure rates of around 80% [3]. However, the success of ATRA-based therapy in AML has not been translated into other non-APL subtypes of AML. Based on in vitro data showing efficacy with cytotoxic chemotherapy agents such as Ara-C and anthracyclines, several clinical studies have investigated the impact of adding ATRA to chemotherapy in patients with non-APL AML [4]. Unfortunately, however, these studies have yielded inconsistent and conflicting results. One possible reason for a lack of response to ATRA treatment in non-APL AML patients is epigenetic repression of the retinoic acid receptor pathway, especially the *RARA* gene [5]. Indeed, combination therapy of ATRA with epi-drugs targeting the histone H3 lysine 4 demethlyase LSD1 (KDM1A) has shown promise in in vivo models of AML [6]. This approach is now being evaluated in a phase I clinical trial where patients are given escalating doses of the LSD1 inhibitor Parnate (tranylcypromine, TCP) with fixed continuous doses of ATRA.

Acute myeloid leukemia is characterized by clonal or oligoclonal hematopoiesis that varies amongst patients. On average, at least four-to-five oncogenic mutations or chromosomal abnormalities are present at diagnosis, along with passenger mutations of unclear significance [7]. Clonal evolution occurs after exposure to chemotherapy, and the secondary mutations that give rise to these clones are more likely to be random, as opposed to recurrent primary abnormalities [8]. Here we report a case of relapsed AML characterized by a previously unidentified chromosomal translocation.

## 2. Results

### 2.1. Clinical Presentation

**Figure 1:**
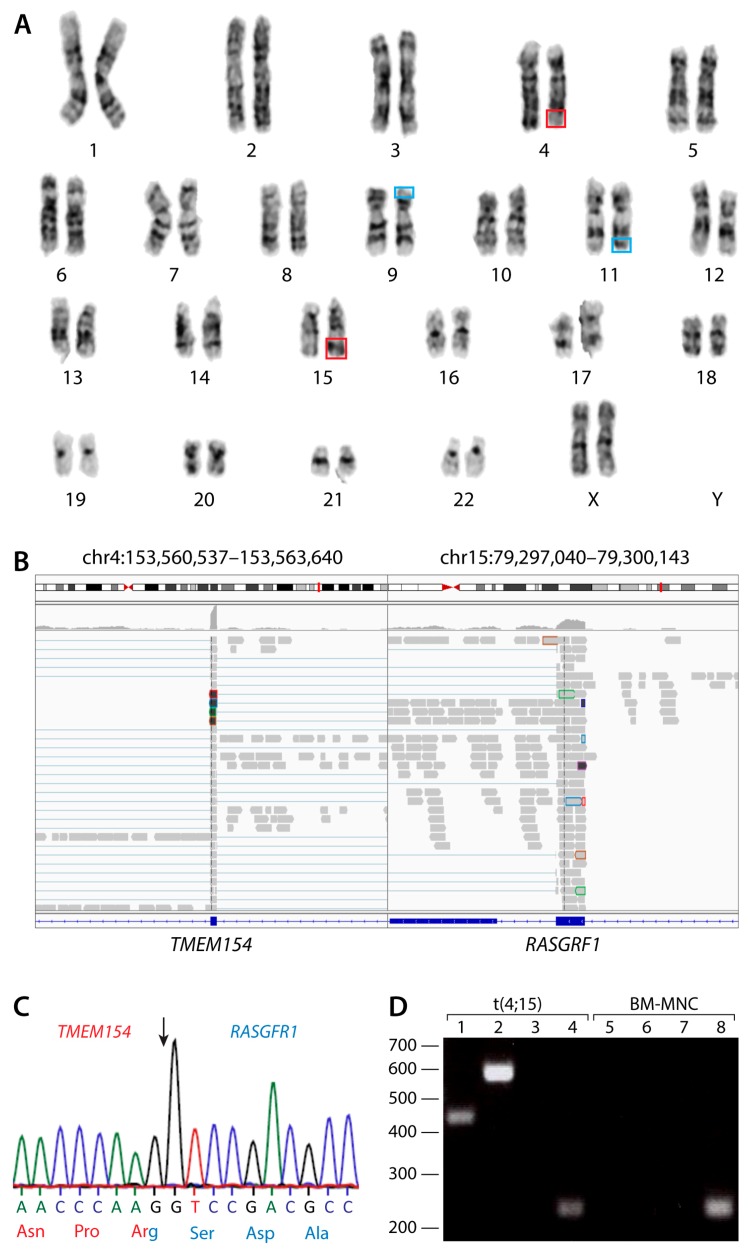
Analysis of the t(4;15)(q31;q22) reciprocal chromosomal translocation. (**A**) Conventional karyotyping showing 46,XX,t(4;15)(q31;q22), outlined in red and t(9;11)(p22;q23), outlined in blue. Chromosome analysis was performed on 20 G-banded metaphase cells from multiple unstimulated cultures. Both translocations were present in all cells examined. (**B**) Breakpoint analysis of the novel fusion transcript produced by the t(4;15) chromosomal translocation. *TMEM154* and *RASGRF1* form a chimeric mRNA transcript with the breakpoint indicated by the black arrow. (**C**) Sanger sequencing chromatogram showing the transcribed sequence surrounding the breakpoint. (**D**) PCR analysis of and *TMEM154*-*RASGRF1*, *RASGRF1-TMEM154*, and *TMEM154* transcripts. PCR was performed on t(4;15) acute myeloid leukemia (AML) cells (lanes 1–4) and bone marrow mononuclear cells (BM-MNCs) from a healthy donor (lanes 5–8). Bands correspond to a 445 bp product amplified from*TMEM154*-*RASGRF1* mRNA (lanes 1 and 5), a 589 bp product amplified from *TMEM154*-*RASGRF1* mRNA (lanes 2 and 6), and a 445 bp product amplified from*TMEM154* mRNA (lanes 4 and 8). No product was amplified using primers corresponding to *RASGRF1-TMEM154* mRNA (lanes 3 and 7).

A 30-year-old previously healthy African-American female presented with a white blood cell count (WBC) of 180,000/µL, hemoglobin 7.6 g/dL, platelets 93,000/µL. Physical examination was unremarkable. Bone marrow aspiration and biopsy were consistent with AML with monocytic differentiation. Blasts amounted to 95% of nucleated elements and expressed CD34, CD117, CD13, CD33, CD64 (weak), CD38, HLA-DR, and were negative for terminal deoxynucleotidyl transferase (TdT) and myeloperoxidase (MPO) by flow cytometry. Cytogenetic analysis revealed an abnormal female karyotype: 46,XX,t(9;11)(p22;q23)[13]/46,XX[7]. Additionally, mutational analysis revealed an activating mutation in *NRAS* gene (c.34G > A; p.G12S). Following treatment with standard therapy, the patient achieved complete cytogenetic remission. Post remission therapy followed, with sequential cycles of high-dose cytarabine in the absence of an optimal donor. Following a disease-free interval of 8 months, the patient relapsed. Karyotype at relapse was consistent with clonal evolution: 46,XX,t(4;15)(q31;q22), t(9;11)(p22;q23)[20] (Figure 1A). Mutational analysis confirmed the presence of an *NRAS* mutation as before. The patient failed two lines of salvage chemotherapy. In the setting of chemotherapy refractory disease, the patient was enrolled in the phase I clinical trial (NCT02273102) and given escalating doses of TCP with fixed continuous doses of ATRA. However, the patient died from rapid disease progression after eight days of treatment (WBC at screening was 400/µL and at withdrawal from study WBC was 99,300/µL).

### 2.2. Molecular and Functional Characterization of the t(4;15) Fusion Gene

To characterize the translocation partners and their breakpoints, we used RNA-Seq and Sanger sequencing, confirming the t(9;11) *MLL*(*KMT2A*)-*AF9*(*MLLT3*) reciprocal translocation present at diagnosis (data not shown). We also defined a novel fusion gene comprising exons 1–6 of the *TMEM154* gene (4q31.3) and exons 15–24 of the *RASGRF1* gene (15q24.2) (Figure 1B). This generates a putative 830 amino acid TMEM154-RASGRF1 fusion protein comprising amino acids 1–178 of TMEM154 and 622–1273 of RASGRF1 (Figure 1C). RT-PCR analysis confirmed the presence of *TMEM154-RASGRF1* mRNA in t(4;15) AML cells but not normal controls (Figure 1D, lanes 1–2 and 5–6). By contrast, controls for *TMEM154* mRNA were present in both t(4;15) AML cells and normal controls (Figure 1D, lanes 4 and 8). Consistent with the RNA-Seq results, expression of mRNA transcripts arising from the reciprocal *RASGRF1-TMEM154* fusion gene were not detected in t(4;15) AML cells (Figure 1D, lane 3).

**Figure 2:**
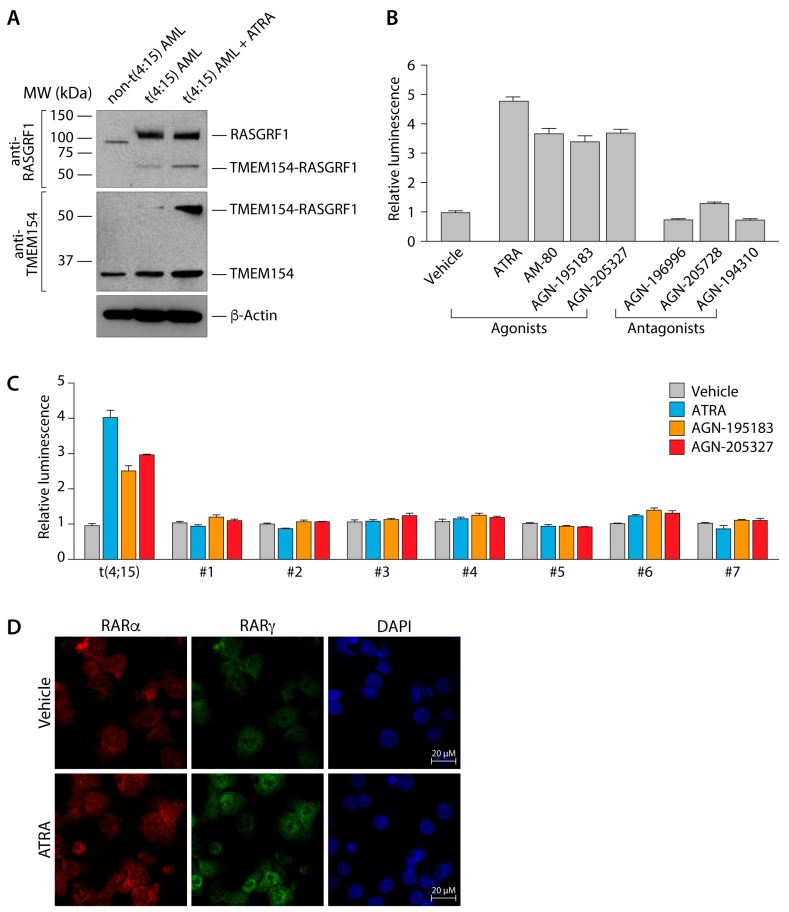
Characterization of t(4;15) AML primary cells. (**A**) Immunoblot analysis of *TEM154-RASGRF1* expression. After 72 h treatment, vehicle control non-t(4;15) AML cells (lane 1), vehicle control t(4;15) AML cells (lane 2) and all-trans retinoic acid (ATRA) t(4;15) AML cells (lane 3) were subjected to immunoblot analysis. Samples were probed with rabbit monoclonal antibody against a C-terminal region of RASGRF1 and mouse monoclonal antibody against an N-terminal region of TMEM154. β-actin was used as a loading control. Molecular weight standards (left) and the identities of bands (right) are indicated. (**B**) Proliferation of t(4;15) AML cells in response to retinoids. Cells were treated with vehicle control, ATRA, RARα agonist (AM-80 or AGN-195183), or RARγ agonist (AGN-205327). Cells were also treated with RARα antagonist (AGN-196996), RARγ antagonist (AGN-205728), or RARα/β/γ antagonist (AGN-194310). All retinoids were used at a concentration of 1 µM. Cell proliferation was determined by CellTiter-Glo luminescent cell viability assay (Promega) after 72 h of treatment. Values shown are normalized to vehicle control. (**C**) Proliferation of t(4;15) and non-t(4;15) AML cells in response to retinoid agonists. Samples from t(4;15) and 7 non-t(4;15) AML patients were incubated with vehicle control, ATRA, RARα agonist (AGN-195183), or RARγ agonist (AGN-205327) at a concentration of 1 µM. Cells were analyzed and normalized as described for (B). (**D**) Treatment of t(4;15) cells with ATRA increases levels of nuclear RARγ. t(4;15) cells were treated with vehicle control or 1 µM ATRA and incubated for 72 h before staining with RARα mouse monoclonal and RARγ rabbit polyclonal antibodies.

In agreement with our analysis of mRNA expression, Western blot analysis revealed the expression of endogenous TMEM154 and RASGRF1 proteins, as well as TMEM154-RASGRF1 fusion protein, which could be detected using antibodies directed against both a N-terminal region of TMEM154 as well as a C-terminal region of RASGRF1 (Figure 2A). Interestingly, treatment with ATRA led to an increase in the expression of TMEM154-RASGRF1 fusion protein, but not endogenous TMEM154 or RASGRF1 (Figure 2A). Supporting the clinical history, we observed a rapid proliferation of the patient’s blasts following treatment with ATRA ex vivo (Figure 2B). The same phenotype was seen when the cells were exposed to other agonists of the retinoic acid pathway. Primary cells lacking a t(4;15)(q31;q22) rearrangement displayed no growth alteration compared to controls when treated with either ATRA (a pan-RAR isotype agonist) or isotype-selective agonists specific for RARα or RARγ (Figure 2C). To further examine the effects of ATRA on primary t(4;15) AML cells, we performed immunofluorescence analysis of RARα or RARγ. Here, we found that treatment of t(4;15) AML cells with ATRA led to an increase in nuclear RARγ (Figure 2D).

## 3. Discussion

Our data suggest that the t(4;15)(q31;q22) reciprocal translocation confers a growth-stimulatory response to retinoid-based therapy. However, as we did not measure levels of apoptosis at the time the proliferation assays were performed, it is possible that t(4;15) cells also exhibited improved survival ex vivo. Nevertheless, an ex vivo growth-stimulatory response is consistent with the increased WBC in the patient following ATRA treatment. The basis for this response requires further investigation, but could lie—at least in part—in an increase in nuclear RARγ following ATRA treatment. While RARα is required for myeloid differentiation, RARγ has been shown to promote hematopoietic stem and progenitor cell self-renewal and proliferation [9]. Additionally, it is likely that the ATRA-mediated increase in TMEM154-RASGRF1 fusion protein (which is consistent with established ATRA regulation of TMEM154 [10]) cooperates with mutant NRAS G12S (which results in decreased GTPase activity and constitutive RAS signaling). Between 12–19% of AML patients possess gain-of-function mutated *RAS* genes [7], with NRAS being most frequently affected [11]. Furthermore, other components of the RAS pathway signaling cascade are often mutated [12]. RASGRF1 (RAS protein specific nucleotide releasing factor 1) is a guanine nucleotide exchange factor (GEF) similar to the *Saccharomyces cerevisiae CDC25* gene product, and functions to activate RAS by catalyzing the exchange of RAS-bound GDP for GTP [13]. While we have not yet investigated the activity of TMEM154-RASGRF1 towards NRAS, it is noteworthy that the fusion protein (which lacks regulatory domains contained in N-terminal RASGRF1) contains the membrane anchoring domain of TMEM154 and functional domains of RASGRF1. These include the GDP/GTP RAS exchanger and CDC25H motifs of RASGRF1, which have been shown to be sufficient for functional protein [14].

Another aspect of the consequences of the t(4;15) translocation that merits further consideration is the fact that *RASGRF1* is a paternally expressed imprinted gene. Thus, under normal conditions, only the paternal allele of the gene is translated into protein. In t(4;15) AML, both the TMEM154-RASGRF1 fusion protein as well endogenous RASGRF1 are expressed, leading to possible gene dosage effects. Conversely, the lack of expression of the reciprocal *RASGRF1-TMEM154* transcript indicates that the differentially methylated domain (DMD) located 30 kb 5′ of the *RASGRF1* transcription initiating site [15] is intact, resulting in imprinted methylation. A functionally important consequence of this could be inappropriate silencing of genes downstream of *TMEM154*. Here, it is noteworthy that the gene immediately downstream of *TMEM154* is *FBXW7*, which encodes an F-box family protein that is a component of the SCF (SKP1-cullin-F-box) ubiquitin protein ligase complex. In the context of AML, FBXW7 has clinically relevant targets including MYC, MCL-1, and Cyclin E [16,17].

## 4. Materials and Methods

### 4.1. Patient Samples

Primary AML samples were collected by the Tissue Banking Core Facility (TBCF) at the University of Miami according to an institutional review board approved protocol (20060858). In order to purify the mononuclear fraction, bone marrow aspirates were subjected to density gradient centrifugation using Ficoll Paque Plus (GE Healthcare Life Sciences, Pittsburgh, PA, USA).

### 4.2. DNA/RNA Isolation

DNA and RNA were isolated from mononuclear cells from patients’ bone marrow aspirate. DNA was isolated using QIAamp DNA Mini Kit (Qiagen, Valencia, CA, USA) according to the manufacturer’s instructions. RNA was extracted with PureLink RNA kit (Life Technologies, Carlsbad, CA, USA) following the manufacturer’s recommendations.

### 4.3. RNA-Seq for Breakpoint Identification

Preparation of RNA libraries for sequencing on the Illumina HiSeq2500 (Illumina, San Diego, CA, USA) platform was carried out in the John P. Hussman Institute for Human Genomics Center for Genome Technology at the University of Miami. Briefly, total RNA was quantified and qualified by Agilent Bioanalyzer (Agilent, Santa Clara, CA, USA) to have an RNA integrity score (RIN) of 7. For each of the two samples, 1000 ng of total RNA was used as input for the Illumina TruSeq Stranded Total RNA Library Prep Kit with Ribo-Zero to create ribosomal RNA-depleted sequencing libraries. Each sample was barcoded to allow for multiplexing and was sequencing to ~30 million raw reads in a 2 × 125 paired end sequencing run on an Illumina HiSeq2500. Raw sequence data from the Illumina HiSeq2500 was processed by the on-instrument Real Time Analysis software (v1.8, Illumina, San Diego, CA, USA) to basecall files. These were converted to demultiplexed FASTQ files with the Illumina supplied scripts in the BCL2FASTQ software (v1.8.4, Illumina, San Diego, CA, USA). The quality of the reads was determined with FastQC software for per-base sequence quality, duplication rates, and overrepresented kmers (www.bioinformatics.babraham.ac.uk/projects/fastqc/). Illumina adapters were trimmed from the ends of the reads using Trim Galore! resulting in two trimmed FASTQ files per sample (http://www.bioinformatics.babraham.ac.uk/projects/trim_galore/). Reads were aligned to the human reference genome (hg19) with the STAR aligner (v2.5.0a) [18]. Gene fusions were detected in the aligned BAM files using the Manta software [19].

### 4.4. Accession Numbers

The Genbank accession number for *TMEM154-RASGRF1* mRNA is MF175878. RNA-Seq FASTQ files were submitted to the sequence read archive (SRA) with the Genbank accession number SRR5681084.

### 4.5. RT-PCR

Reverse transcription and PCR amplification was performed using One-Step RT-PCR System (Life Technologies) according to manufacturer’s instructions. Primers used to amplify *TMEM154-RASGRF1* mRNA were *TMEM154*-Fwd333: 5′-TAGCCAAGGATCTCAGAGTG-3′; and *RASGRF1*-Rev778: 5′-GAATGGCACTGATAGGCTTC-3′ or *RASGRF1*-Rev922: 5′-GTGACGATGTCTTGGTGATG-3′. Primers used to amplify TMEM154 were *TMEM154*-Fwd1762: 5′-TAGCCAAGGATCTCAGAGTG-3′ and *TMEM154*-Rev2272: 5′-TGAGCGCCATTCAGGTTTAG-3′. Primers to amplify *RASGRF1-TMEM154* were RASGRF1-Fwd1762: 5′-CTCATTCAGGTGCCCATGTC-3′ and *TMEM154*-Rev2272: 5′-TGAGCGCCATTCAGGTTTAG-3′. Amplified PCR fragments were subjected to DNA sequence (Eurofins, Louisville, KY, USA) to validate the translocation breakpoint.

### 4.6. Western Blot

Protein sample concentrations were normalized using DC Protein Assay (Bio-Rad, Hercules, CA, USA). Fifty micrograms of protein were separated by SDS-PAGE using 4–20% Mini-PROTEAN TGX pre-cast gels with Precision Plus ProteinDual Color Standards (Bio-Rad). Primary antibodies used were as follows: rabbit monoclonal antibody against the C-terminal region of RASGRF1 (ab118830, Abcam, Cambridge, UK) and mouse monoclonal antibody against the N-terminal region of TMEM154 (sc-398802, Santa Cruz Biotechnology, Dallas, TX, USA). β-actin was used as a loading control (A5441, Sigma, St. Louis, MO, USA,). Protein bands were detected using HRP-linked Anti-rabbit IgG (#7074, Cell Signaling Technology, Danvers, MA, USA) and HRP-linked Anti-mouse IgG (Cell Signaling Technology, #7076) secondary antibodies.

### 4.7. Cell Proliferation

Cell proliferation was measured using CellTiter-Glow luminescent cell viability assay (Promega, Madison, WI, USA) following the manufacturer’s recommendations. Primary AML mononuclear cells were seeded at a density of 10,000 cells/well in 96-well plate for 24 h, followed by retinoid or vehicle (DMSO) treatment for 72 h. Cell were cultured in RPMI supplemented with 5% Charcoal-dextran fetal bovine serum (FBS, Life Technologies). ATRA was purchased from Sigma. AM-80 was purchased from Tocris (Bio-Techne, Minneapolis, MN, USA). AGN195183, AGN196996, AGN205327, AGN205728, and AGN194310 were manufactured under contract at the Chinese National Center for Drug Screening, Shanghai Institute of Materia Medica, Shanghai, China.

### 4.8. Immunofluorescence

AML cells were fixed with 4% paraformaldehyde in 1X phosphate-buffered saline (PBS) for 20 min at room temperature. For immunostaining, cells were permeabilized with 1% Triton X-100 in 1X PBS and blocked with 2.5% bovine serum albumin (BSA) and 1% Triton X-100 in 1X PBS for 1 h shaking at room temperature. Primary antibodies (dilution 1:100) were incubated for 2 h at room temperature in blocking buffer and washed five times with wash buffer (0.1% Triton X-100 in 1X PBS). Primary antibodies used were RARα-9a (mouse monoclonal) and RARγ-453 (rabbit polyclonal) (gift from Cécile Rochette-Egly, L’Institut de Génétique et de Biologie Moléculaire et Cellulaire (IGBMC), France). Secondary antibodies (dilution 1:1000) were added for 1 h at room temperature in blocking buffer and then washed five times. Secondary antibodies used were Alexa Fluor 488-conjugated anti-mouse IgG (H + L), F(ab’)2 fragment (Cell Signaling Technology, #4408) and Alexa Fluor 555-conjugated anti-rabbit IgG (H + L), F(ab’)2 fragment (Cell Signaling Technology, #4413). Negative controls were performed using primary and secondary antibodies alone. Slides were then mounted with ProLong Gold antifade reagent with DAPI (Molecular Probes, Eugene, OR, USA), following the manufacturer’s instructions. Immunofluorescence microscopy was performed using a Leica DFC 310 FX microscope.

## 5. Conclusions

Here we report a case of relapsed AML characterized by a novel translocation associated with aggressive disease and increased proliferation in the presence of ATRA. We are currently generating a cell line bearing this translocation that will provide a new model system for the study of AML. Although the *TMEM154-RASGRF1* translocation is likely to be a rare occurrence (as has been the case in the past with other translocations), it is predicted that further study of its biology will yield important insights into retinoic acid signaling and resistance to retinoid-based therapy in AML.
